# Emerging roles of bacteriophage-based therapeutics in combating antibiotic resistance

**DOI:** 10.3389/fmicb.2024.1384164

**Published:** 2024-07-05

**Authors:** Anandhalakshmi Subramanian

**Affiliations:** Department of Microbiology and Parasitology, College of Medicine, King Khalid University, Abha, Saudi Arabia

**Keywords:** antibiotic resistance, bacteriophage-based therapeutics, synergistic effect, preclinical and clinical studies, formulation strategies, advantages over antibiotics

## Abstract

Amid the growing challenge of antibiotic resistance on a global scale, there has been a notable resurgence in bacteriophage-based treatments, signaling a shift in our approach to managing infections. Bacteriophages (BPs), bacterial predators of nature, present a promising alternative for tackling infections caused by antibiotic-resistant pathogens. This review delves into the intricate relationship between bacteriophages and resistant bacteria, exploring various treatment strategies. Drawing upon both preclinical and clinical studies, the review highlights the effectiveness of bacteriophage therapy, particularly when integrated synergistically with conventional antibiotics. It discusses various treatment approaches for systemic and localized infections, demonstrating the adaptability of bacteriophage therapy across different clinical scenarios. Furthermore, the formulation and delivery of bacteriophages shed light on the various methods used to encapsulate and administer them effectively. It also acknowledges the challenge of bacterial resistance to bacteriophages and the ongoing efforts to overcome this hurdle. In addition, this review highlights the importance of the bacteriophage sensitivity profile (phagogram), which helps tailor treatment regimens to individual patients and specific pathogens. By surpassing the limitations of traditional antibiotics, bacteriophage-based therapies offer a personalized and potent solution against antibiotic resistance, promising to reshape the future of infectious disease management.

## 1 Introduction

Antibiotics play a vital role in curing bacteria-caused minor and life-threatening infections. Nonetheless, bacteria have become incredibly resilient and are capable of using numerous approaches to survive the attack by most antibiotics (Roach and Donovan, 2015). Globally, there is a growing rate of antibiotic resistance (AR) and the rise of multidrug-resistant superbugs, largely caused by the non-judicious usage of antibiotics. In addition to jeopardizing healthy living, AR has a huge economic impact on both developed and developing countries (Aslam et al., 2021). As per the World Health Organization (WHO), AR is responsible for causing approximately 700,000 deaths every year. Moreover, the number of AR-caused deaths is estimated to rise rapidly in the upcoming years (Ling et al., 2022). On average, patients suffering from AR-associated infections require approximately 13 days of hospital stay, imposing a cost of approximately $29,000 per patient treated for such resistant bacterial infections. This can lead to a total annual potential productivity loss of approximately $35 billion (Khurshid et al., 2017; Aslam et al., 2018; Khurshid et al., 2020). These statistics indicate the global impact of AR and the urgency to find novel and effective antimicrobial therapies to control the AR crisis (Baloch et al., 2018; Aslam et al., 2021; Ling et al., 2022).

Bacteriophages (BPs) or phages are viruses that are natural bacterial killers. Approximately, an incredible number (10^31^) of BPs are present in the world, which outnumber bacteria by an estimated tenfold (Skurnik, 2022). In recent times, BP therapy has gained a lot of attention as a powerful and potential therapeutic antibacterial approach (Taati Moghadam et al., 2020; Wei et al., 2020; Tamma and Suh, 2021). BPs are more specific to their targets than antibiotics, and therefore, BPs can eradicate the target pathogenic bacteria without altering the commensal microflora. On the other hand, broad-spectrum antibiotics kill pathogenic bacteria in infected individuals and disrupt microbes and normal bacterial flora (Rai and Kumar, 2022). Antibiotic-resistant bacterial pathogens are also accountable for causing severe nosocomial infections, particularly in individuals with life-threatening conditions. Furthermore, various pathogens can escape the activity of antibiotics by hiding in safe niches within the host; for instance, *S. aureus* has the ability to endure in the intracellular environment including abscesses or biofilms, which leads to persistent infections. The deficiency of new antibiotics and the rise of multidrug-resistant bacterial strains necessitate the development of alternative therapies, for example, BP-based therapies (Schmelcher and Loessner, 2021).

This review aims to explore the potential of BPs as an alternative to antibiotics in combating antimicrobial resistance. In addition, the advantages of BP-based therapies over antibiotics, phage-antibiotic synergy, the potential of antimicrobial BP-derived proteins, multiple formulation strategies of BP therapy, preclinical and clinical progress of BP-based therapeutics against clinically significant pathogens, and factors that need to be considered in BP therapy have been highlighted in this review.

## 2 Antibiotic-resistance crisis

**Figure 1:**
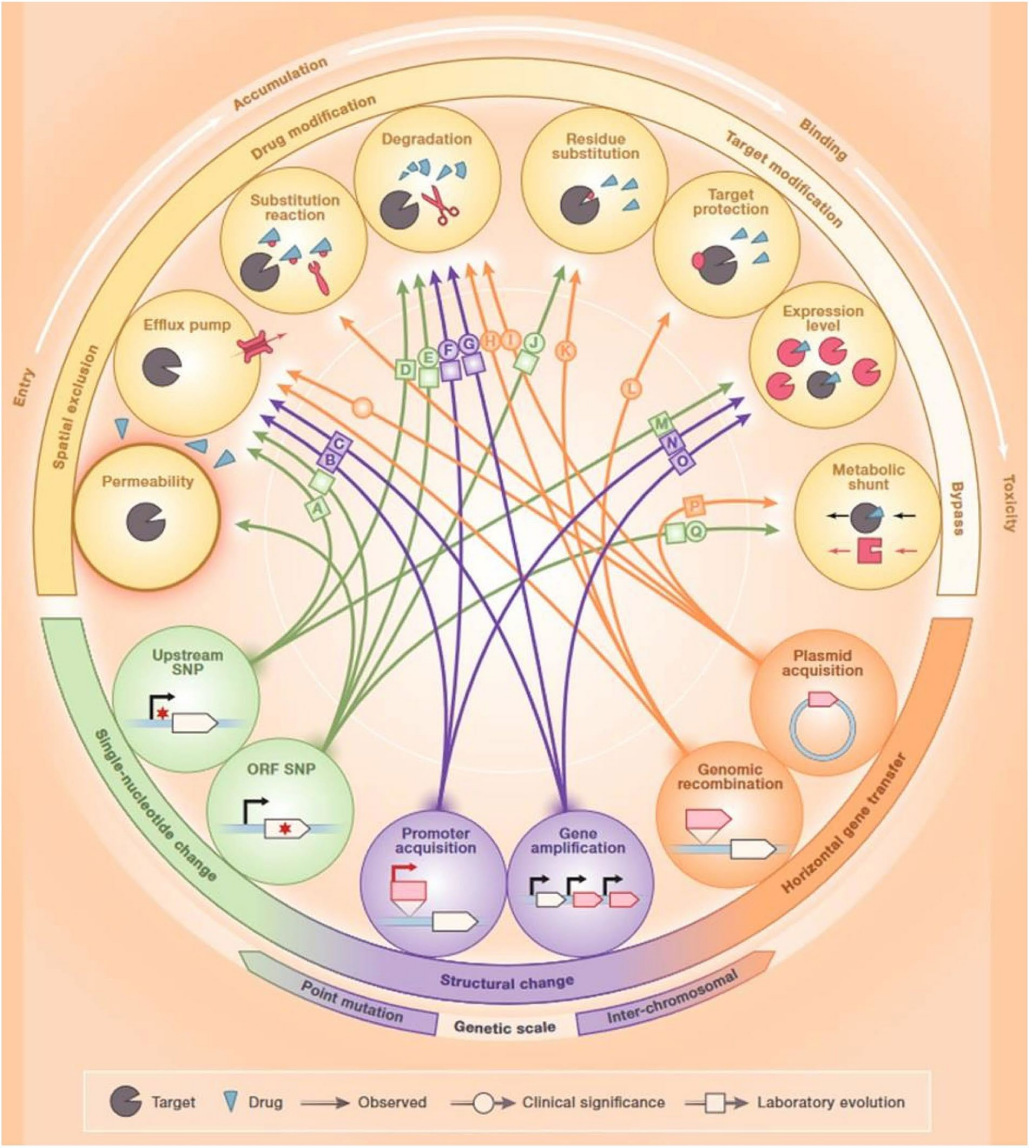
Mechanisms of antibiotic resistance. Reproduced with permission from Elsevier (Yelin and Kishony, 2018).

Antibiotic resistance (AR) is a global phenomenon that even precedes the development and usage of antibiotics by human beings. Microorganisms can generate numerous biologically active molecules across diverse environments that possess antibacterial properties; some of these molecules have been further developed and are currently used as antibiotics. The levels of these molecules within natural environments are frequently under the clinically relevant thresholds, which indicates that resistance does not solely occur to evade their toxic activity. In nature, the interplay between antibiotics and AR mechanisms plays the role of a communication channel between the microbial community members instead of only antimicrobial agents. In addition, these molecules induce genotypic and phenotypic responses and shape the composition of the community. Therefore, genes conferring AR to modern antibiotics were also detected in ancient microbial communities (Perron et al., 2015). Along with the treatment of human infectious diseases, antibiotics are also utilized to mediate livestock growth and treat fish and crop diseases in aquaculture and agriculture (Ventola, 2015). As high as 180 mg of active antibiotic agent is used per kilogram of produced meat in the United States, an even higher proportion has been reported in other countries (Price et al., 2017; Gordillo Altamirano and Barr, 2019). A major problem of using antibiotics in agriculture is the release of millions of tons of antibiotics into environmental reservoirs and water effluents (Manyi-Loh et al., 2018). A lack of proper water treatment facilities further exacerbates this problem. Moreover, pharmaceutical waste and reduced distance between cities and farmlands also play a role in elevating the extents of antibiotic release and environmental persistence. Indeed, this continuous introduction of environmental microorganisms to a range of antibiotics has resulted in increased evolution and extended the range of AR genes in natural reservoirs. AR can also occur due to the genetic information transfer between organisms through transduction, conjugation, or transformation (Yelin and Kishony, 2018) (Figure 1).

Antibiotic-resistant bacteria found in the guts of animals exposed to antibiotics are a major source of horizontal transfer determinants. In addition, bacterial cells can attain non-genetically encoded, transient resistance through various mechanisms including persistence, metabolic dormancy, swarming adaptation, and growth in biofilms (Xu et al., 2022). Numerous resistance mechanisms can hinder the effectiveness of antibiotics at every step of their passage through the bacterial cell. First, bacteria can modify the structure of their cell envelope to repudiate the entry of the antibiotics or can generate efflux pumps to expel antibiotics (Yelin and Kishony, 2018) (Figure 1). Bacteria can inhibit the production of enzymes needed for the activation of antibiotics, or they can also destroy or alter antibiotics by producing enzymes including β-lactamases. Bacteria can hide, modify, or quantitatively alter the intended targets of the drug. Finally, bacteria can cause the activation of alternative metabolic signaling pathways to evade the toxic effects of antibiotics (Munita and Arias, 2016). Since antibiotic-resistant bacteria are rising at an alarming rate, alternative therapies are urgently required for the control of bacterial infections (Alaoui Mdarhri et al., 2022).

## 3 Bacteriophages

Bacteriophages (BPs) are viruses that can infect bacterial hosts (Skurnik, 2022). Typically, BPs share all the common features of viruses, and they cannot replicate by themselves. They need bacterial hosts for reproduction. BPs are small (50–200 nm), and they transmit genetic instructions for effective and fast replication. Generally, BPs are specific to a particular bacterial host; any one BP might infect multiple different species within a genus and many or most strains within a species. However, occasionally, it may affect very few or only one specific isolate of a species (Aslam et al., 2021). Several BPs infect bacteria from diverse genera and usually can do so only when they are phylogenetically closely associated (Tamma and Suh, 2021). The host range of BP can be broad (numerous distinct species or even distinct genera) or very narrow (where only a limited number of bacterial isolates assist replication). Indeed, the host preference or host range largely determines the therapeutic potential of BPs (Rai and Kumar, 2022).

### 3.1 Replication cycles of bacteriophages

#### 3.1.1 Lytic cycle

**Figure 2:**
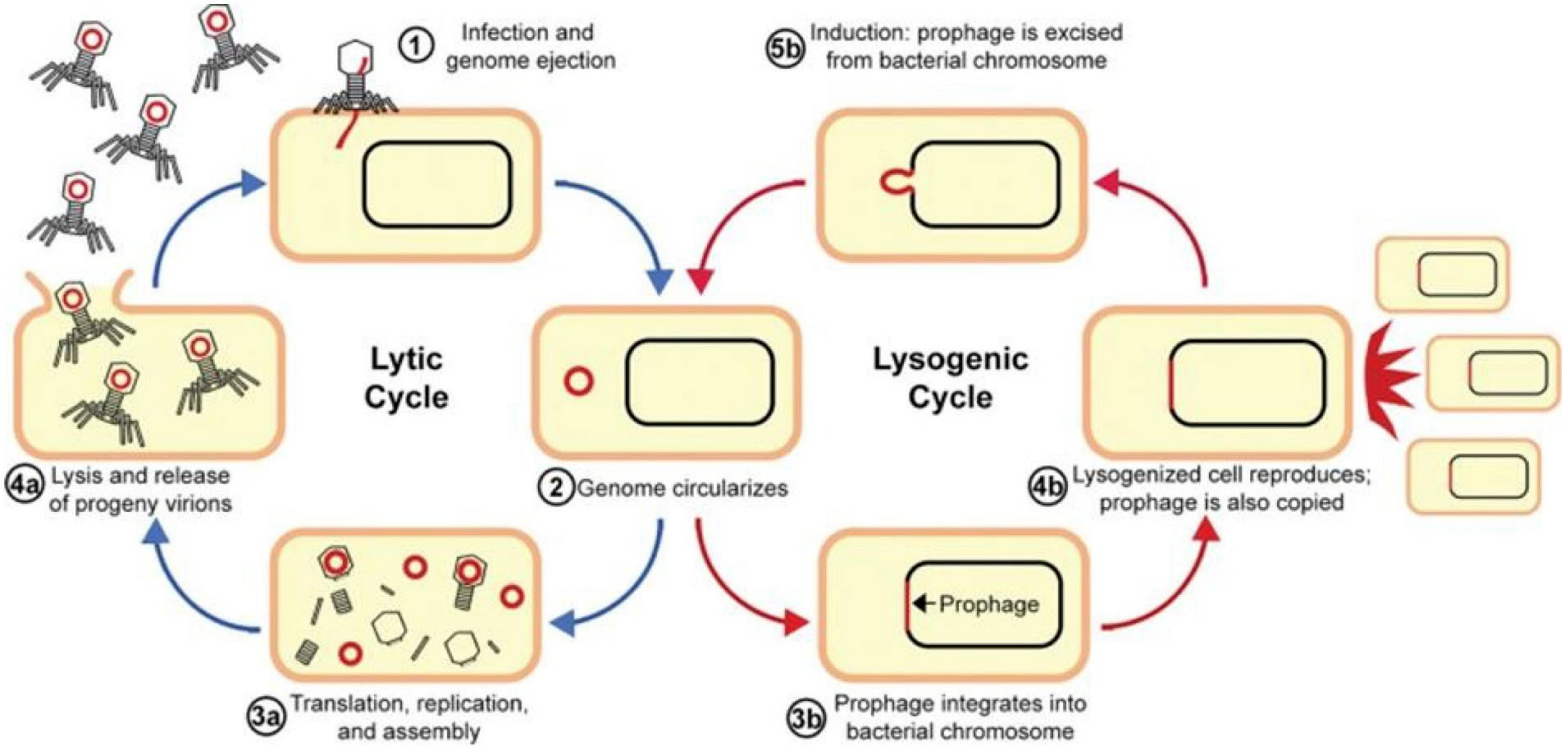
Replication cycles of bacteriophages. Reproduced with permission from Elsevier (Alaoui Mdarhri et al., 2022).

A BP delivers the genomic content into a bacterium during a lytic infection cycle by attaching with the receptor(s) on its surface, and subsequently, it goes through viral replication in the cytosol through bacterial transcription, translation, and replication. After the generation of new BP particles, they evade through the cytoplasm by causing bacterial lysis (Figure 2). This mechanism is subsequently repeated by the new BP particles as they cause infection of additional susceptible cells. It has been reported that this mechanism plays a beneficial role in BP therapy, where lytic BPs are used as self-amplifying drugs that can locate and eradicate susceptible cells, which might be more effective as compared with antibiotics, since antibiotics are not capable of self-amplification (Kortright et al., 2019).

#### 3.1.2 Lysogenic cycle

In the case of lysogeny or a lysogenic cycle, BPs incorporate their nucleic acids into the chromosomes of a cell instead of causing cell lysis. Furthermore, viral proteins integrate viral DNA into the bacterial chromosome; as a result, the virus exists as prophage DNA, which is replicated each time the cell divides. It has been observed that in some cases, viral prophages are maintained as circularized; small *plasmids* of DNA within the cell are also stably inherited. A single infected cell in this way can rapidly propagate the BP DNA without leading to cell lysis (Louten, 2022).

## 4 Bacteriophage-based therapies

### 4.1 Polymeric bacteriophages

Various conditions including food poisoning, colorectal cancer, ulcerative colitis, and Crohn’s disease can disturb the intestinal flora. The upper digestive tract primarily contains Gram-positive facultative anaerobes and bacterial concentration in this tract at a rate of 10^3^–10^4^ CFU/mL. Nonetheless, in the colon, this concentration can increase up to 10^11^–10^12^ CFU/mL, which mostly contains various anaerobic bacteria including *Enterobacter*, *Enterococcu*s, *Clostridium*, *Eubacterium, Bifidobacterium*, and *Bacillus*. Most of the studies on polymer-encapsulated BPs have explored their potential in treating gastrointestinal tract (GIT) infections. Various factors regarding GIT need to be considered when developing oral BP formulations for animal or human use, such as the impact of various digestive ferments (including trypsinogen, amylase, lipase, protease, and pepsin), harsh pH conditions, effects of bile salts, and varying retention times of different intestinal segments, including the ileum and duodenum. The main goal of polymeric encapsulation is to protect BPs from the adverse gastric environment to evade inactivation and reduction of BP titer (Silva Batalha et al., 2021). Indeed, polymeric carriers could be developed to withstand various pH values, for instance, pH of small intestine (pH 5.5–6.5), stomach (pH 1–3), and colon (pH 6.5–7.2). Several chemically modified biopolymers (such as polyamino acids, polycarbonates, polyamides, and aliphatic polyesters) are available commercially and licensed for preparing their coatings or microspheres (Ling et al., 2022). Alginates are widely utilized to encapsulate various BPs for oral delivery. Cross-linked gels are formed upon exposure of alginate to two valence cations, and extrusion (a physical encapsulation method) can be used for microencapsulation. Further addition of calcium carbonate in alginate gel beads can significantly enhance the acid-resistance capacity of encapsulated BPs (Colom et al., 2017). Polymer-encapsulated BPs with anti-acid agents are more stable in acidic environment than free BPs. Whey protein, chitosan, pectin, guar gum, and neutral gum can also be utilized to ameliorate the stability of alginate microparticles in acidic conditions. The gut flora can generate a range of substances such as β-xylosidase, urea dehydroxygenase, nitroreductase, β-galactosidase, α-arabinosidase, and β-glucosidase to use the undigested substrate in the small intestine. Multiple enzyme-sensitive polymers such as pectin-, methylcellulose-, hydroxypropyl-, and cellulose-derived biopolymers can react with the aforementioned enzymes and release on the target site, which can eventually result in the target site regulation of specific flora (Colom et al., 2017; Ling et al., 2022).

### 4.2 Bacteriophage cocktails

It is essential to exactly match the pathogens and BPs in BP-based therapies. Nonetheless, *in vivo* lysis properties and *in vitro* screening of BPs might not always match. Sometimes BP monotherapy is not adequate to fulfill the clinical requirements, even though BPs have the capacity to overcome antibiotic-induced AR. Therefore, a BP cocktail-based therapy is important to overcome the limitation of the narrow antibiogram of BP monotherapy. BP cocktails can be used to target multiple strains or a single bacterial strain of a single bacterial species, or even several bacterial species. Nevertheless, the purification and preparation processes of such cocktails are complex due to their complex composition. Moreover, the pharmacodynamic and pharmacokinetic properties of BPs derived from cocktails are erratic (Monteiro et al., 2019). Thus, the relative efficacy of each cocktail constituent should be assessed, and BPs with low-efficacy ought to be discarded (Abedon, 2017). Interestingly, BP cocktails could be further designed in a way that each BP is sequentially active, rather than being active simultaneously with the other BPs in a cocktail. Henceforth, even if resistance occurs because of continuous administration, new non-resistant BPs will continue to exert their effects. Studies with animal models demonstrated that the sequential BP cocktail administration can result in outstanding results in overcoming BP resistance and decreasing bacterial populations. BP cocktails are already available in Georgia and Russia as over-the-counter medicines to treat bacterial infections. Pyo BP developed by Eliava biopreparations is also used to treat suppurative and intestinal diseases caused by *Proteus mirabilis, Streptococcus pyogenes, Pseudomonas aeruginosa*, *Proteus vulgaris,* and *Escherichia coli* (Ling et al., 2022) [Eliava Institute of Bacteriophage (Russia), Microbiology and Virology in Tbilisi (Georgia) Microbiology].

### 4.3 Liposome-encapsulated bacteriophages

The major two problems of the low therapeutic efficacy of oral BPs include inadequate retention time in the intestine and poor stability of BPs in gastric acid environment, which requires repeated administration of free BPs. There are several disadvantages of frequent or repeated administration including expensive, time-consuming, and poor patient compliance (Singla et al., 2016). These problems can be avoided by using positively charged liposomes. Liposomes can act as proton barriers and provide protection to BPs from gastric acids (Otero et al., 2019). Furthermore, the intestinal retention time of BPs can also be extended because of their positively charged surfaces (Cui et al., 2017; Kuerban et al., 2020). Enterobacteria BPs encapsulated in cationic liposomes were developed in a study conducted by Colom et al. (2017).

There are several advantages of cationic liposome-loaded BPs because of their positive charge on their surfaces including enhanced mucus adhesion and prevention of inactivation of BPs in gastric acid conditions, which further extends the intestinal residence time of BPs (Ma et al., 2016). Moreover, smaller particles of liposome-encapsulated BPs can enhance the chances of cell uptake in the host cytoplasm via membrane fusion or endocytosis (Ma et al., 2016; Fukuta et al., 2017), which can then inactivate various intracellular pathogens. Functionalization of liposomes is also possible to increase the clinical applications of BPs by altering the charge distribution of liposomes to extend the retention time, encapsulating multiple probes to observe the pharmacokinetics of liposome-loaded BPs, enhancing the circulation time *in vivo,* delaying the release of contents, and attaching specific targeting ligands to the surfaces of liposomes. Nonetheless, liposomes can attach and even fuse under specific conditions, which is undesirable for clinical applications and storage (Nieth et al., 2015; Ling et al., 2022).

### 4.4 Microneedle platform for transdermal delivery of bacteriophages

Oral BPs have also been tested as a topical treatment against infectious gut microbiota. Typically, BPs do not enter into systemic circulation from the GIT. Moreover, intestinal carbohydrates and bile salts might block the bivalent metal ions that are essential for the replication of BPs. Thus, oral BPs encounter challenges in achieving a systemic therapeutic effect. On the other hand, parenteral administration of BPs is a more efficient way to permit BPs to exhibit a systemic therapeutic role. Intravenous administration of BPs has some drawbacks including poor compliance, maintenance of a costly cold chain, and chances of cross-contamination. However, these aforementioned problems can be solved by microneedle-mediated transdermal delivery of BPs. In this regard, a hollow polycarbonate microneedle array has been developed by the researchers. The bottom diameter and average height of this device were 750 μm and 995 μm, respectively, and the hollow aperture was 100 μm. It was observed that this device can fully perforate the skin and deliver BPs. Bioavailability can reach up to 100% through this transdermal delivery device. The width and depth of the residual skin pores are 600 μm and 210 μm, respectively, which can be closed rapidly following treatment. In addition, the functions of the epithelial barriers are not disrupted in this process. Nonetheless, it is hard for microneedles to transport stock solutions through all skin layers. It has been reported that microneedles do not have the capacity to cross all skin layers, resulting in a liquid pool on the skin surface (Ling et al., 2022).

### 4.5 Electrospun biopolymer fiber-encapsulated bacteriophages

In recent times, there has been growing interest in electrospun biopolymer fibers as delivery devices or reliable carriers for bioactive agents. BPs can also be fixed internally or wrapped on their surfaces (Korehei and Kadla, 2014; Díaz et al., 2018). There are several advantages of producing fibers via the electrospinning method, such as porosity, larger surface area, softness, and flexibility. Thus, these fibers can be used locally in the form of packaging materials or wound dressings and bandages with antibacterial properties. In addition to targeting inactivated bacterial pathogens, the bioactive surfaces are also effective in BP-mediated immobilization, identification, and detection of targeted microbes. It is possible to achieve a controlled release of BP particles with electrospun fibers by selecting appropriate materials. Interestingly, the controlled release kinetics of BPs can also be achieved by mixing different fiber polymers with different molecular weights (Ling et al., 2022).

## 5 Advantages of bacteriophage-based therapies over antibiotics in the treatment of infections

In general, antibiotics can act against a wide range of microbial species and therefore can be used to treat a broad range of bacterial pathogens. However, numerous bacteria have already developed many mechanisms for obtaining AR. In this regard, genetic alterations including horizontal gene transfer (HGT) and mutation also play a role. In addition, various physiological factors including eradication of the antibiotics and enzymatic modification can play a role in altering efflux pump and cell permeability (Munita and Arias, 2016). Several resistance mechanisms developed by bacteria can be passed to others via HGT. In contrast, distantly related bacteria (non-host) are not likely to have ever been exposed to the BP of interest because of the host specificity of BPs. As a result, these bacteria are not likely to play a role in resistance genes to non-hosts through HGT (Cohan et al., 2020). Similarly, CRISPR-based BP immunity is unlikely to protect other taxa and therefore does not play a role in spreading resistance through HGT (Cohan et al., 2020). Thus, the probability of spread and development of BP resistance is markedly lower than that of the development of AR. Moreover, a synergistic effect can be achieved by the combined use of BPs and antibiotics (Gu Liu et al., 2020), since targeted bacterial strains struggle to simultaneously counter two different types of stress (Burmeister et al., 2020; Khan et al., 2022). A comparative advantage of BP-based therapies over antibiotics in the treatment of infections is presented in Table 1.

**Table 1:** Advantages of bacteriophage-based therapies over antibiotics in treating infections.

	Bacteriophages	Antibiotics	References
Anti-biofilm activity	Effectively penetrate and destroy biofilms	Less effective against biofilms	Tian et al. (2021)
Specificity to bacteria	High degree of strain or species specificity	Non-specific and broad spectrum of activity	Chegini et al. (2021)
Disruption of beneficial microbiome	Limited impact on beneficial microbiome	Disrupts beneficial microbiome	Patangia et al. (2022)
Dosing	Self-amplifying in target bacteria after the initial administration and increased concentration at the target site	Constant dosing is required to maintain inhibitory concentrations	Principi et al. (2019)
Discovery process	Fast	Slow	Osman et al. (2023)
Resistance	Bacteriophage can co-evolve to infect resistant bacteria, therefore limited chances of developing resistance	More recurrently utilised to treat infections, therefore more possibility of developing resistance	Koderi Valappil et al. (2021)
Impact on the immune system	Negligible impact on the immune system exerted by highly purified bacteriophage preparations	Exert direct impact on the immune system by numerous immunomodulatory antibiotics	Van Belleghem et al. (2019)
Environmental impact	Shorter life outside host, which results in fast eradication from the environment	Release in the environment can lead to waterbody contamination, which can further contribute to antibiotic-resistance	Khan et al. (2022)
Adverse effect	Side effects linked with bacteriophage therapy have rarely been reported	Various side effects are observed along with the treatment	Opperman et al. (2022)

## 6 Phage-antibiotic synergy (PAS)

**Figure 3:**
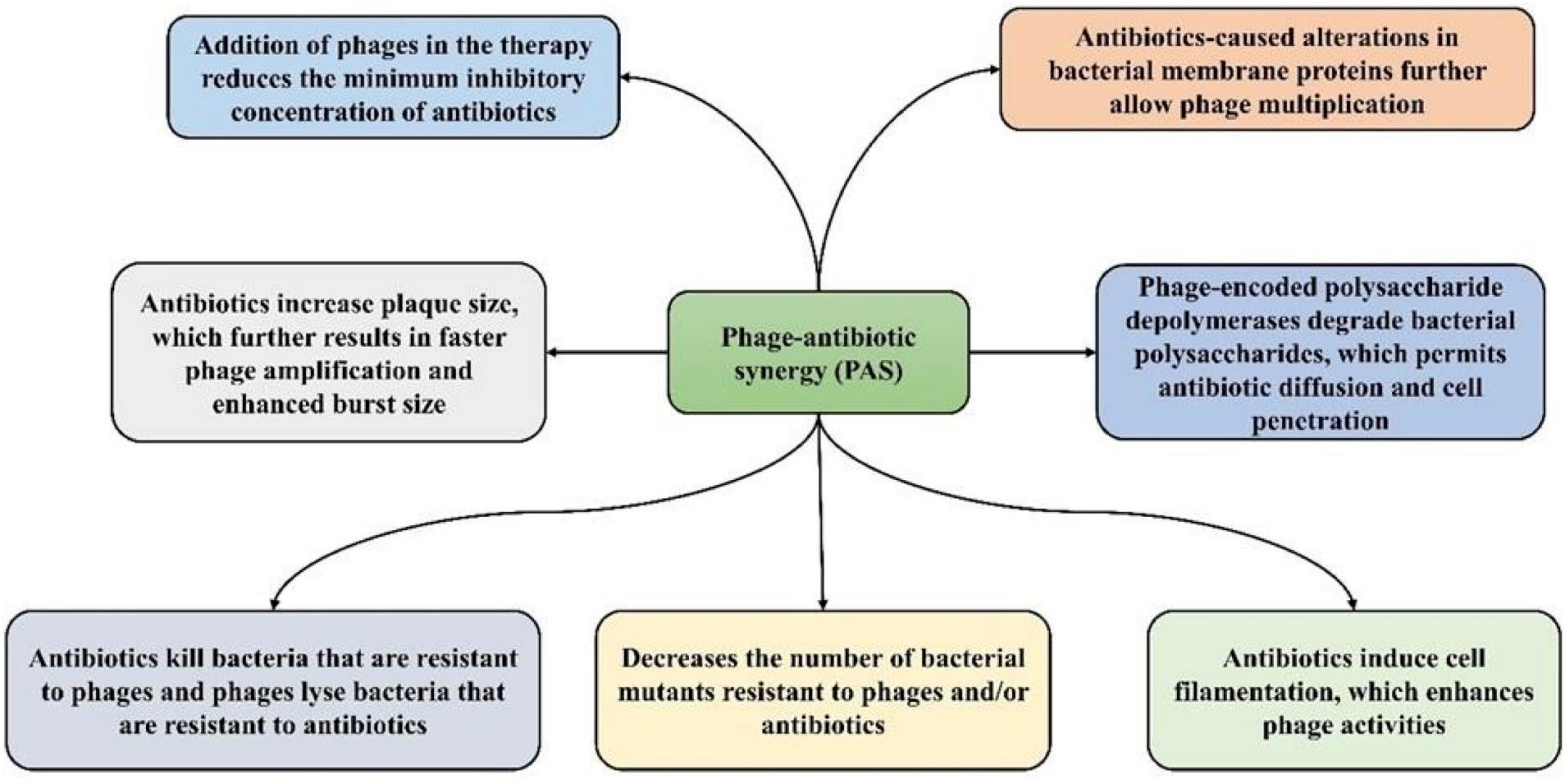
Potential mechanisms of phage-antibiotic synergy (PAS).

Phage-antibiotic synergy (PAS) is defined as the phenomenon where the combined effect of BPs and antibiotics is greater compared with the sum of either BPs or antibiotics alone (Chaudhry et al., 2017; Simon et al., 2021). It has been reported that certain antibiotics induce the generation of BPs via a bacterial host and form larger plaques (Figure 3) in the occurrence of antibiotics (Kamal and Dennis, 2015; Li et al., 2021). In addition, the release of progeny BPs from bacterial cells can occur due to the sublethal concentrations of some antibiotics. PAS can decrease the concentration of antibiotics utilized in therapy and ultimately decrease the emergence of AR in bacterial populations (Jo et al., 2016b). The killing mechanism of bacteria differs between BPs and antibiotics (Chaudhry et al., 2017). PAS involves several mechanisms that enhance phage production and/or antibiotic efficacy. Antibiotics such as β-lactams and quinolones induce cell filamentation, potentially aiding phage lysis enzyme (Knezevic et al., 2013). Some antibiotics, such as linezolid and tetracycline, increase plaque size and phage amplification, while others such as cefotaxime enhance phage burst size leading to higher phage concentrations (Ryan et al., 2012).

Subinhibitory antibiotic concentrations inhibit the emergence of phage and/or antibiotic-resistant mutants (Kebriaei et al., 2020). Phage therapy can sensitize bacteria to antibiotics, lowering the MIC and increasing susceptibility (Gu Liu et al., 2020). BPs can also degrade bacterial polysaccharides, aiding antibiotic diffusion. These mechanisms collectively underscore the potential of combined phage-antibiotic therapies against drug-resistant infections. Therefore, it has been suggested that BPs ought to be combined with antibiotics for even better control of bacteria than BPs alone (Torres-Barceló and Hochberg, 2016). The use of BPs and antibiotics is predominantly suggested for infections caused by Gram-positive bacteria such as multidrug-resistant *Enterococcus* strains and methicillin-resistant *Staphylococcus aureus* (MRSA). Nonetheless, in terms of killing *S. aureus*, PAS was found to be effective against the antibiotic-susceptible strains compared to the antibiotic-resistant ones (Jo et al., 2016a), which further indicates that antibiotics can cause alterations in bacterial membrane proteins that can cause alteration in the BP receptors. In a study, Simon et al. showed synergism between the lytic *S. aureus* BP Sb-1 at BP multiplicity of infection of 10^−1^ and 10 and oxacillin at concentrations varying from 5 to 100 μg/mL for most studied isolates of *S. aureus*. The combination of oxacillin and BP Sb-1 markedly reduced the level of bacteria than oxacillin alone (Simon et al., 2021). A study conducted by Malik S showed that combining an *E. coli* phage cocktail with antibiotics reduced MIC values, notably from 8 to 2 μg/mL for amikacin and from 32 to 8 μg/mL for fosfomycin. These findings underscore how phages serve as adjuvants, bolstering antibiotic effectiveness against drug-resistant *E. coli* strains (Malik et al., 2021).

Daptomycin (an antibiotic) is often used to treat infections caused by *Enterococcus faecium*, since 80–90% of *Enterococcus faecium* strains are resistant against ampicillin and vancomycin. It has also been reported that PAS synergy including daptomycin was linked with lower BP resistance. In another study, Morrisette et al. (2022) observed bactericidal activity with *E. faecium* BP cocktail and daptomycin in most regimens. In addition, daptomycin introduced to the BP averted BP resistance against daptomycin-resistant *E. faecium* (Łusiak-Szelachowska et al., 2022). Elevated BP titers and larger plaques were noticed when used in increasing antibiotic concentrations.

A study observed that the combination of low-dose meropenem and *B. cepacia* BP elevated the survival rate of *Galleria mellonella* larvae (Kamal and Dennis, 2015). In another study, Aghaee et al. used a combination of antibiotics and BPs against the *P. aeruginosa* strain PA14 isolated from a burn (Aghaee et al., 2021). This strain was treated with antibiotics at a sub-minimum inhibitory concentration (MIC) and MIC levels, a combination of BPs, a mixture of two BPs, and a single BP. Subsequently, the four lytic BPs were selected based on their performance in a primary efficiency of plating test. Moreover, out of these four primarily sequenced BPs, those with different infection properties and genetic features were selected. Interestingly, all designated BPs formed plaques on *P. aeruginosa*. Collectively, these findings suggested that a combination of one antibiotic and two BPs showed the highest killing potential against the strain of *P. aeruginosa*. In another study involving *Citrobacter amalonaticus*, synergistic effects were observed with the BP MRM57 at concentrations of 10^3^ and 10^6^ plaque forming units (PFU/ml), combined with a sublethal dose of antibiotics with a distinct mode of action (including tigecycline, cefepime-tazobactam, meropenem, gentamicin, fosfomycin, colistin, and carbenecillin) at 1/10 × MIC (Łusiak-Szelachowska et al., 2022; Manohar et al., 2022).

## 7 Antimicrobial bacteriophage-derived proteins

BP genomes encode several important enzymes and proteins to eradicate bacteria during infection. Polysaccharide depolymerizing enzymes (PDEs) and virus-associated peptidoglycan hydrolases (VAPGHs) are two such groups of proteins which are required for BPs to adsorb with and infect host bacteria (Roach et al., 2017). VAPGHs are typically found in the outermost layer of the protein shells of BPs, which is a component of the viral structure and plays a role in the local peptidoglycan layer degradation so that the tail tube structures of BPs release their genomes into the host. BP-encoded PDEs can cause the degradation of polysaccharide components found on the bacterial cell membrane surface, for instance, lipopolysaccharides derived from Gram-negative bacteria. Furthermore, PDEs also help BPs reach the host receptors by digesting the polysaccharides present on the bacterial cell membrane, which can further degrade biofilms (Maciejewska et al., 2018). There is a growing interest regarding BP-derived lysins or cell wall hydrolases as potential alternatives to antimicrobials. Lysins are also considered promising clinical candidates because of their differential virulence, low drug resistance, and rapid bactericidal action (Sharma et al., 2018).

BP-derived lysins possess enzymatic functions and can destroy non-divided bacterial cells, which can be a therapeutic option against recurrent or drug-resistant infections. Ectolysins and endolysins are the two major lysine enzymes. Ectolysins play a role in decomposing peptidoglycans externally to bacteria during BP DNA invasion. Endolysins also decompose the peptidoglycans of the cell wall at the end of the replication of BPs (Schmelcher et al., 2012). Various lysine enzymes such as amidases, peptidases, and glycosidases can split distinct chemical bonds according to the selectivity of lysins. The addition of a small quantity of lysin can rapidly decrease the optical density of turbid-suspended Gram-positive bacterial cultures (>10^8^ CFU/mL) within minutes. Moreover, lysine does not exert any effect on eukaryotic cells since murein hydrolases are only present in bacteria. For instance, p128, a chimeric recombinant exolysin derived from BP K, completely inactivated prokaryotic cells and did not exhibit any cytotoxicity on eukaryotic cell lines including Hep 2 and Vero even at a high concentration of 2.5 mg/mL (Ling et al., 2022). The biofilm and capsule structure of bacteria play an important role in resistance that averts the actions of antibiotics. The capsule structures of numerous bacteria can assist pathogens to escape phagocytosis and mediate cell invasion, intravascular survival, and epithelial colonization. Similarly, the biofilm structure can protect the bacterial population against immune functions. Thus, it is possible to remove or target these structures by using BP-derived depolymerases. Moreover, it was confirmed that the synergistic effect of amoxicillin and depolymerase could destroy the biofilms of *Klebsiella pneumoniae* (Ling et al., 2022).

## 8 Preclinical and clinical progress of bacteriophage-based therapeutics against clinically significant pathogens

### 8.1 Preclinical studies using animals

#### 8.1.1 Respiratory infections

In mouse models, Waters et al. reported the full removal of a cystic fibrosis strain of *P. aeruginosa* in a study, where two BP doses were intranasally administered to infected mice 48/60 or 24/36 h following infection. Interestingly, after providing treatment at 144/156 h post-infection, a significant reduction was observed in 30% of mice, while complete eradication was observed in 70% of mice. It was also reported that BP therapy markedly ameliorated the survival rate of *CF*-like lung disease in mice following intranasal administration 2 h post-infection. Administering a higher dose of BP 4 days before the bacterial challenge completely protected the mice, which further suggests that prophylactic therapy with BP can avert chronic infections (Waters et al., 2017). In a different study, Semler et al. assessed various routes of BP delivery in a murine model of *Burkholderia cepacia* complex respiratory infection. Bacterial load was reduced by 100-fold when BP was administered through nebulization, whereas no reduction was detected following intraperitoneal administration (Semler et al., 2014). Collectively, these findings regarding curative and prophylactic treatment of respiratory infections with BP suggest that respiratory infections might be a potential target for effective BP therapy (Kortright et al., 2019).

#### 8.1.2 Gastrointestinal infections

Therapies with BP in the treatment of GIT bacterial infections can significantly decrease or even prevent virulent bacteria colonization without affecting the natural gut microbiota. Four days following an adherent-invasive *E. coli* challenge, Galtier et al. (2017) noticed that a preventative BP treatment averted the advancement of colitis symptoms and decreased colonization of bacteria in the gut of mice treated with dextran sodium sulfate. Prophylactic therapy with BP administered 2 h before bacterial challenge led to a 100% mortality rate in an insect model of *Clostridium difficile* colonization, whereas coadministration of BP and bacteria resulted in a 72% reduction in the mortality rate. Moreover, BP treatment 2 h after bacterial challenge led to a 30% reduction in the mortality rate (Nale et al., 2016). In the small intestine of infant mouse models, Yen et al. (2017) reported that prophylactic therapy with a BP cocktail decreased colonization of *Vibrio cholerae* when BPs were provided 3 and 6 h before bacterial challenge. Nonetheless, BP-resistant bacterial mutants were recovered following treatment, and reduced outcomes were observed with BP therapy when taken over 6 h prior to bacterial challenge as well as when a higher dose of *V. cholerae* was used to challenge mice (Yen et al., 2017). Indeed, the findings of the prophylactic therapy of GIT infections with BP are promising; however, more studies are required on the treatment after bacterial challenge, since prophylactic therapies are always not feasible in clinical settings (Kortright et al., 2019).

#### 8.1.3 Localized infections

BP therapy for a number of localized infections such as infected burns, urinary tract infections, and otitis has a great potential to completely avoid the usage of chemical antibiotics. Moreover, the usage of chemical antibiotics for hospital-acquired and surgical infections is often limited, since this usage can result in strains with extreme AR. Several studies reported positive outcomes in using bacteriophages (BPs) for infected ulcers (Fish et al., 2018; Morozova et al., 2018). Notably, integrating BPs into biodegradable wound dressings with sustained antimicrobial release, as demonstrated by Kvachadze et al. (2011), showed promising results. The capacity of BP to treat an *E. coli*-caused urinary tract infection was investigated in a murine model. Interestingly, 48 h after BP therapy, BP administered through the intraperitoneal route after 24 h of bacterial challenge reduced the bacterial load by 100-fold in the kidneys after 48 h of BP therapy (Dufour et al., 2016). Treatment with the same BP markedly reduced the bacterial load in an *E. coli* pneumonia model; however, the treatment was not effective in an *E. coli* model of sepsis (Kortright et al., 2019).

#### 8.1.4 Systemic infections

Various studies have already assessed the effectiveness of BP in treating systemic infections. In a study, Alemayehu et al. reported the efficacy of two phages (φNH-4 and φMR299-2) in combating clinical *Pseudomonas* strains in murine lungs and biofilm environments, bolstering the appeal of phage therapy as a strategy for managing multidrug-resistant Pseudomonas lung infections in *CF* patients (Alemayehu et al., 2012). In another study, the administration of a single intraperitoneal injection of the recently discovered phage EF-P29 showed effectiveness against vancomycin-resistant *Enterococcus faecalis* (VREF) strains, successfully combating bacteremia in murine models. This highlights the considerable potential of phage therapy in managing systemic VREF infections (Nasr Azadani et al., 2020). Multiple factors determine the favorable outcome of BP in the treatment of systemic infections, which needs to be rigorously assessed before broader usage of BP in sepsis treatment in humans (Kortright et al., 2019).

#### 8.1.5 Combination therapy of bacteriophages and antibiotics

A study conducted by Fernando L et al. investigates the effectiveness of combining phage therapy with antibiotics against *Acinetobacter baumannii* infections. Using a murine model, researchers found that the combination treatment (PBS, phage øFG02, and ceftazidime) significantly reduced bacterial burden compared to controls or single treatments. Interestingly, phage (phage øFG02) therapy alone led to bacterial resistance, but these resistant strains became more susceptible to antibiotics. This suggests that phage therapy can sensitize bacteria to antibiotics, offering a promising strategy against antibiotic-resistant infections (Altamirano et al., 2022). On the other hand, no mortality was observed with the combination therapy of BP and enrofloxacin. In a similar study, Oechslin et al. reported that treatment with a combination of BP and ciprofloxacin led to a 10,000-fold decrease in bacterial load in comparison with the ciprofloxacin or BP treatment alone in rats with experimental *P. aeruginosa* endocarditis. Moreover, it was observed that the combination of BP and ciprofloxacin led to synergistic *in vitro* and *in vivo* killing of *P. aeruginosa* (Oechslin et al., 2017). Since combined BP therapy with chemical antibiotics has great potential, more studies are required to reveal the possible synergy between BP and antibiotics (Jennes et al., 2017; Hoyle et al., 2018; Kortright et al., 2019).

### 8.2 Clinical studies

#### 8.2.1 Skin and soft tissue infections (SSTIs)

Eight clinical trials registered on clinicaltrials.gov focus on evaluating bacteriophage therapy for SSTIs, predominantly in phase I or phase I/II stages. Atopic dermatitis, venous leg ulcers, pressure ulcers, and diabetic foot ulcers are all included in these trials (Hitchcock et al., 2023).

A clinical trial has evaluated the effectiveness and safety of BPs in the treatment of burn wounds infected by *P. aeruginosa* (Jault et al., 2019). In this trial, a cocktail of 12 BPs with lytic action against *P. aeruginosa* was introduced to an alginate template that was directly applied to the wound, while a topical application of 1% sulfadiazine silver was used in the control group. Moreover, the average sterilization time for the control group and BP-treated group was 47 h and 144 h, respectively. It was observed that these unpredictably poor outcomes could occur owing to the administration of a lower BP dose than envisioned. A dose of 200–2,000 PFU was utilized rather than the expected 2 × 10^7^ PFU dose, and the level of the BP cocktail dropped throughout the study. It was concluded that the lack of effectiveness of the therapy could be due to the unintended alteration in the treatment protocol (Kortright et al., 2019).

Another study, REVERSE, assessing a five-phage cocktail targeting multiple bacteria, showed promising safety results with efficacy data pending (Technophage, SA, 2022). The WPP-201 trial, a phase I study for venous leg ulcers, concluded that the phage therapy was safe, but no phase II efficacy study has been identified (Wolcott, 2011). These clinical trials represent significant steps in evaluating the efficacy and safety of bacteriophage therapy for various skin and wound infections. While some trials have shown promising safety results, efficacy data are still pending for many.

#### 8.2.2 Diarrheal diseases

A clinical trial was conducted by Sarker et al. (2016) to evaluate the effectiveness and safety of two different cocktails of BP that target pathogenic *E. coli* in diarrheal diseases. In this trial, only the individuals exhibiting acute onset of dehydrating diarrhea were included. The BP therapy involved oral administration of either a T4-like BP cocktail (T) consisting of 11 BPs and a commercial BP cocktail (M) from Russia containing at least 17 different BPs in oral rehydration solution, while the placebo group received a standard treatment with an oral rehydration solution. Any significant differences and adverse effects were not observed between the placebo and BP therapy groups. Furthermore, it was not clear whether the BPs showed lytic activities against the exact strains of *E. coli* responsible for causing the disease, and no further actions were taken before the BP administration to buffer the stomach. Thus, a substantial number of BP particles were possibly not able to endure the low-pH environment of the stomach, and the surviving particles were not able to increase because of the deficiency of a proper host (Sarker et al., 2017).

#### 8.2.3 Bloodstream infections

Only a few ongoing clinical trials focus on bacteremia and phage therapy. One of these trials is a phase I study assessing the safety, tolerability, and pharmacodynamics of three different doses of an *E. coli* phage genetically modified to deliver a CRISPR/Cas payload (SNIPR001). Participants receive the treatment twice a day for 7 days. The second trial, DiSArm, is a phase II study investigating the safety, tolerability, and efficacy of *S. aureus*-specific phage cocktail (AP-SA02) alongside antibiotics for individuals with *S. aureus* bacteremia (SNIPR, 2023; Armata Pharmaceuticals, Inc, 2024a).

#### 8.2.4 Lung infections

The majority of the seven ongoing clinical trials target lung infections associated with cystic fibrosis (*CF*), such as those brought on by *Pseudomonas aeruginosa*. The Cystic Fibrosis Foundation is backing these trials since *P. aeruginosa* has a major negative effect on the health of *CF* sufferers. There are three *CF* trials recruiting subjects (Koff, 2023). The MUCOPHAGES experiment, which ended in April 2012, looked at how 10 bacteriophages target pseudomonas-affected 59 *CF* patients’ induced sputum. No results, though, have been made public (University Hospital, Montpellier, 2013). The same product, an inhaled *P. aeruginosa* phage cocktail known as AP-PA02, is the subject of two lung-targeted phage trials called Tailwind and SWARM-Pa. Phase II trials Tailwind and SWARM-Pa are seeking volunteers with chronic *P. aeruginosa* infections and non-*CF* bronchiectasis (Armata Pharmaceuticals, Inc, 2024b).

#### 8.2.5 Otitis

Wright et al. conducted a clinical trial to evaluate the preliminary effectiveness and safety of BP therapy in treating *P. aeruginosa-*caused chronic otitis*. In this trial,* a cocktail of 6 BPs with lytic properties was used to treat individuals with *P. aeruginosa*-caused chronic otitis (Wright et al., 2009). A marked cumulative reduction in the bacterial counts was observed in the BP treatment group, while no marked cumulative alteration was observed in bacterial counts in the placebo group. Interestingly, by day 42, three participants from each group experienced undetectable *P. aeruginosa* levels. In the BP treatment group, BPs were obtained for an average of 23 days from participants, which suggested that bacteria became BP resistant, BP was not able to reach the infection site, or BPs were cleared following the resolution of the infection. The BP therapy was found to be safe in otitis treatment, since no severe adverse events were observed in either group (Wright et al., 2009). A summary of various clinical and preclinical findings on the potential of BP-based therapeutics against infections is shown in Table 2.

**Table 2:** A summary of clinical and preclinical findings on the potential of bacteriophage-based therapeutics against infections.

Condition	Causative agent	Used bacteriophage	Route of administration	Study model	Study outcome	References
**Preclinical studies**						
*Vibrio parahaemolyticus* infection	*Multiple-antibiotic–resistan Vibrio parahaemolyticus*	Phage pVp-1	Intraperitoneal and oral	Mice	92% (intraperitoneal) and 84% (oral) reduction of mortality were observed in BP-treated mice through	Jun et al. (2014)
Bacteremia	*Imipenem-resistant P. aeruginosa*	Phage Ø9882	Intraperitoneal	Mice	100% survival in the treatment group	Wang et al. (2006)
Sepsis	*Pseudomonas aeruginosa*	Phage strain KPP10	Oral	Mice	66.7% reduction of mortality in the bacteriophage (BP)-treated group	Watanabe et al. (2007)
Ileocecitis	*Clostridium difficile*	*C. difficile* specific phage CD140	Oral	Hamster	Most of the BP treated hamsters survived, while all the control animal died within 96 h after challenge	Chanishvili (2012)
Bacteremia	β*-lactamase producing Escherichia coli*	Phage Ø9882	Intraperitoneal	Mice	100% survival in the treatment group	Wang et al. (2006)
Meningitis and Sepsis	*Escherichia coli*	EC200^PP^ (a lytic phage)	Intraperitoneal or subcutaneous	Rat pups	50 and 100% reduction of mortality in case of sepsis and meningitis, respectively	Watanabe et al. (2007)
Wound infection	*Staphylococcus aureus*	Phage LS2a	Subcutaneous	Rabbit	Administration of BP and *S. aureus* prevented staphylococcal infection	Wills et al. (2005)
**Preclinical trials**						
Wound infections	*Pseudomonas aeruginosa*	Anti-*Pseudomonas aeruginosa* bacteriophages (PP1131)	Topical	Human	BP treatment reduced bacterial burden in burn wounds	Jault et al. (2019)
Typhoid	*Salmonella typhi*	typhoid bacteriophages	Oral	Human	Five-fold reduction of typhoid cases in the treated group	Kutateladze and Adamia (2008)
Diabetic foot ulcer	*MDR S. aureus*	Staphylococcal phage Sb-1	Topical	Human	All six treated patients recovered following BP applications	Fish et al. (2016)
Dysentery	*Shigella dysenteriae*	Cholera bacteriophage	Oral	Human	All treated individuals recovered after 24 h following a single administration	Chanishvili (2012)
Chronic Otitis	Antibiotic-resistant *P. aeruginosa*	Biophage-PA	Oral	Human	Significantly lower level of *P. aeruginosa* was reported in the BP-treated group, along with no adverse event	Mardiana et al. (2022)
Cholera	*Vibrio cholerae*	Cholera Bacteriophage	Oral	Human	93% survival rate was observed in the BP-treated group versus 37% survival rate in the control group	Chanishvili (2012)

#### 8.2.6 Urinary tract infections

In a systematic review of clinical trials, bacteriophage administration resulted in significant microbiological improvements, with 62% of articles reporting complete bacterial eradication or urine sterilization (Al-Anany et al., 2023). Additionally, clinical symptom improvement was observed in 97% of human patients treated with bacteriophages. The trials also reported low rates of relapse or re-infection post-treatment, with only 0.8% experiencing re-infection after 2–4 months. This suggests that bacteriophage therapy may offer durable protection against UTIs. Some studies investigated combination therapy involving both bacteriophages and antibiotics. These trials showed enhanced efficacy compared with bacteriophage therapy alone, highlighting the potential synergistic effects of combining these treatments (Terwilliger et al., 2021).

Six clinical trials evaluate the use of phage preparations to treat infections in the genitourinary tract, focusing mainly on asymptomatic UTIs, with one trial targeting bacterial vaginosis. Among these trials, two have been completed. In the first completed trial, Pyo phage, a commercially available treatment from the Eliava Institute in Georgia, was assessed for its efficacy in treating urinary tract infections in patients undergoing prostate transurethral resection. Participants were randomly assigned to receive intravesicular Pyo phage, standard care, or placebo bladder irrigation. The study found that the phage treatment was comparable to standard care or placebo, but low treatment titers may have affected efficacy (Leitner et al., 2017, 2021). The second completed trial, a phase Ib study, examined a CRISPR-Cas3-enhanced phage cocktail targeting *E. coli.* This trial focused on understanding the pharmacokinetics, pharmacodynamics, and safety of the treatment when administered via the intraurethral route. Currently, one of the ongoing trials is recruiting participants for phase II/III trials to assess the efficacy of different doses of the same phage cocktail for acute uncomplicated UTIs caused by multi-drug-resistant *E. coli* in female participants (Kim et al., 2021). These trials highlight the potential of phage therapy in treating genitourinary tract infections, emphasizing the importance of factors such as phage concentration at the infection site and pathogen load in designing effective treatment regimens.

## 9 Factors to be considered in bacteriophage therapy

### 9.1 Bacteriophage-mediated control of biofilm

Biofilm formation is a crucial virulence factor in the case of most MDR pathogens. BPs have been extensively evaluated for their roles against bacterial biofilms. Several studies reported that specific BPs can cause the degradation of biofilms of *S. aureus*, *K. pneumoniae*, *E. coli*, and *Acinetobacter baumannii* (Lin et al., 2017; Dakheel et al., 2019; Wintachai et al., 2020; Osman et al., 2023). This property has been credited to the generation of multiple hydrolases in the capsid that can successfully disturb and disperse biofilms of bacteria (Lin et al., 2017). Interestingly, some BPs possess tail fibers along with inherent depolymerase functions (Kvachadze et al., 2011). However, more studies are required regarding the mechanisms and roles of BPs in disturbing biofilms (Carascal et al., 2022).

### 9.2 Bacteriophage formulation

Typically, the storage medium was mixed with therapeutic BPs to generate a formulation of BP. BP formulations ought to be prepared according to the recognized quality assurance standards and current good manufacturing practices like any other therapies. Moreover, BP formulations should be free from impurities and contaminants including host cell proteins and endotoxins (Jennes et al., 2017; Furfaro et al., 2018; Lehman et al., 2019). As the delivery routes largely determine the effectiveness of BP treatment, several studies have evaluated distinct routes of administering BPs for therapeutic purposes. Topical application for burn and skin infections is a common method of administering BP therapy (Morozova et al., 2018). Oral administration is another mode of administering BPs. Since BPs will be taken internally, BPs need to be stable at human physiological pH (4–9) and body temperature (35–39°C). Various other factors such as bile salts and low gastric pH also need to be considered while formulating BPs. Thus, BP formulations can be enclosed in a gel or include bicarbonate water (Kakasis and Panitsa, 2019). In this regard, in chickens, chitosan nanoparticles loaded with BP ΦKAZ14 were used as an alternate approach to oral delivery. Researchers also suggested that the *in vivo* stability of chitosan-BP nanoparticles was higher than standalone BP (Kaikabo et al., 2016). In chicken, this nanoparticle also regulated the bacterial colonization of avian pathogenic *E. coli* and reduced the colibacillosis symptoms (Kaikabo et al., 2017). Various other routes of BP administration include the systemic route via intramuscular or intraperitoneal injection (Criscuolo et al., 2017; Jennes et al., 2017), the respiratory route via intranasal spray (Casey et al., 2018), and rectal route via fecal microbiota transplant. However, these routes are yet to be demonstrated clinically or *in vivo* (Carascal et al., 2022).

### 9.3 Bacteriophage resistance

Bacteriophage (BP) resistance is another important factor that needs to be carefully considered in BP therapy. It is assumed that the evolutionary pressure exhibited by the BP markedly surpasses any resistance mechanisms gained by the target pathogen (El-Shibiny et al., 2017; Harper, 2018). Moreover, the fast evolution of BPs helps in ameliorating their virulence toward the target pathogen, which makes adaptive bacterial resistance ineffective to BPs. Nonetheless, the existence of BP-resistant bacteria is unavoidable and ought to be considered while developing BP-based therapies (Casey et al., 2018). In Singapore, a small colony variant of *P. aeruginosa* was found to be resistant to PB1 (a BP) has been described in Singapore (Lim et al., 2016); however, its susceptibility to other BPs is yet to be explored. Researchers should consider the bacterial mechanisms against BPs following their administration in order to prevent BP resistance. Such mechanisms might involve abortive infection, superinfection exclusion, and CRISPR-Cas system. BP cocktails also could be considered to avert the rising BP resistance (Carascal et al., 2022).

### 9.4 Pharmacodynamic properties

As with antibiotics, it is essential to determine the pharmacodynamic properties of BPs to substantiate an efficient dosage regimen for the effective control of a range of severe infections. An appropriate BP dosage is required to confirm that an adequate level of virus is present in the infection site to encounter the target pathogen (Zhang et al., 2018). A higher level (10^8^–10^10^) of BPs is administered to the patients (typically in a single-dose regimen) in an inundated therapy to destroy numerous target pathogens (Danis-Wlodarczyk et al., 2021). However, some studies have revealed that a higher titer of BPs involves a risk of causing BP-mediated lysis of non-targeted bacterial cells. This can lead to disturbances in the delicate balance of the commensal gut microbiota, which plays a crucial role in maintaining health. However, in a study, it was significantly less when compared to the broad-spectrum antibiotic activity on the microbial flora. Indeed, further studies are required to establish this finding (Barbu et al., 2016; Cieplak et al., 2018).

### 9.5 Phagogram profiling

Not all BPs can be used for therapeutic purposes. In addition, therapeutic BPs ought to be lytic to confirm the destruction of the target bacteria (Lehman et al., 2019). The BP also ought to have a short generation time, species-specific action, and an increased rate of adsorption to the target bacteria (Khawaja et al., 2016). As with antibiotics, a BP equivalent of an antibiogram or profiling of the BPs in a phagogram is also required (Barbu et al., 2016). In a phagogram, the BP efficacy is continuously tested against a well-defined collection of pathogens known as a diversity panel or a pathogen library (Casey et al., 2018). The phagogram profiling can make sure that the therapeutic BPs are specific to their target pathogen (Abdulamir et al., 2015; Jennes et al., 2017; Furfaro et al., 2018; Phothichaisri et al., 2018; Dakheel et al., 2019; Tan, 2020; Rahimi-Midani et al., 2021; Chung et al., 2023).

## 10 Challenges and opportunities in phage therapy and the future of phage research

Bacteriophage therapy to treat bacterial infections encounters several obstacles and opportunities in terms of applicability and future development. As mentioned earlier, these include bacteriophage formulation, understanding its pharmacodynamic properties, the development of phage resistance in bacteria, and finally ethical and legal considerations (Zalewska-Piątek, 2023).

Optimizing the administration of bacteriophage preparations is crucial to maximizing their therapeutic potential. Understanding the pharmacokinetics of phages, including their stability, distribution, and clearance in the body, is essential for determining the most effective dosage regimens and administration routes (Dąbrowska, 2019). Encapsulation technologies, such as microencapsulation or liposome encapsulation, can protect phages from degradation in the gastrointestinal tract and enhance their bioavailability. Finally, advancing clinical experience and monitoring adverse reactions are paramount for refining phage therapy protocols and ensuring patient safety (Loh et al., 2021). Real-time observation of treatment outcomes, coupled with rigorous data collection and analysis, can provide valuable insights into the efficacy and safety of phage therapy in diverse clinical settings. Phage therapy necessitates the use of lytic phages, ensuring high purification, as lysogenic BPs are unable to lyse host bacteria and also hinder the lytic action of other phages upon integration. Moreover, they pose a significant concern as they can transfer toxins and antibiotic-resistance genes to bacteria (Carascal et al., 2022).

Regarding bacterial resistance to BPs, some bacteria develop defense techniques against phages, such as inhibiting surface receptors, altering mechanisms, and secreting extracellular polymeric capsules. These defenses can impair the capacity of bacteriophages to efficiently infect and destroy bacteria (Wang and Leptihn, 2024). Furthermore, BPs have a narrow cleavage spectrum which limits their effectiveness against different bacterial strains. This suggests that some bacteriophages may only be effective against a restricted group or species of bacteria, rendering them ineffective against a wide range of pathogens (Chung et al., 2023). Hence, the emergence of bacterial resistance mechanisms to bacteriophages emphasizes the need for novel techniques. Using a bacterial cocktail, which includes several phage types that target different bacterial strains, can improve treatment efficiency while decreasing the possibility of resistance development (Rahimi-Midani et al., 2021). Genetic engineering of BPs would increase their host range or boost their lytic activity against certain bacteria (Sun et al., 2023).

Additionally, ethical and legal issues encompass various aspects such as patient safety, informed consent, regulatory approval, and research ethics. The strategy is to establish a comprehensive phage bank and streamlined screening protocols to isolate phages that target antibiotic-resistant bacteria effectively (Anomaly, 2020; Zalewska-Piątek, 2023).

It facilitates the swift matching of BPs to bacterial isolates from infected patients, allowing for personalized treatment. Globally, only a few centers, such as those in Georgia, Belgium, Poland, the United States, and Australia, persist in offering phage-based treatments. These centers offer valuable data on therapy efficacy and conduct clinical trials to further enhance treatment outcomes (Zalewska-Piątek, 2023).

The objective is to eliminate bacterial infections in patients via cell lysis while minimizing adverse effects and preserving the natural microflora.

## 11 Conclusion

The growing global crisis of AR has inspired the development of BPs as an alternative to antibiotics. Indeed, BPs have great potential as effective, promising, and novel therapeutic agents to fight against AR. Despite the aspirations and novelty of the usage of BPs as an alternative to antibiotics, more studies are required to explore the clinical applications of these therapeutic BPs. As antimicrobial resistance is a global issue now, it is crucial to explore the potentiality of BPs as an effective alternative to antibiotics. However, more preclinical and clinical studies are required on the site-specificity, dosage regimens, half-life, and safety profile of the BPs to demonstrate their safety, efficacy, and potential as alternatives to antibiotics.

## Author contributions

AS: Conceptualization, Methodology, Project administration, Formal Analysis, Writing – review & editing, Writing – original draft, Funding acquisition.
